# Beefing up communication skills of upper-level animal science students

**DOI:** 10.1093/tas/txae007

**Published:** 2024-01-12

**Authors:** Shannon L Norris-Parish, Holli R Leggette, Theresa Pesl Murphrey, Jean A Parrella, Audra Richburg, Andy D Herring

**Affiliations:** Department of Agricultural and Extension Education, New Mexico State University, Las Cruces, NM 88003, USA; Department of Agricultural Leadership, Education and Communications, Texas A&M University, College Station, TX 77843, USA; Department of Agricultural Leadership, Education and Communications, Texas A&M University, College Station, TX 77843, USA; Department of Agricultural, Leadership, and Community Education, Virginia Tech, Blacksburg, VA 24060, USA; Department of Agricultural Leadership, Education and Communications, Texas A&M University, College Station, TX 77843, USA; Department of Agricultural Leadership, Education and Communications, Texas A&M University, College Station, TX 77843, USA

**Keywords:** beef cattle experience, beef production course, communication skills, curriculum development, workforce development

## Abstract

Animal scientists face an increasing need to communicate with the lay public because of the public’s interest in the origin and production of animal-sourced foods. Consumers’ increased interest infers a critical need for effective communication skills among animal science graduates. Effective communication skills are mandatory if students are to explain scientific information and mitigate misinformation about livestock production. The purpose of our study was to investigate the communication styles and communication effectiveness of upper-level animal science students enrolled in a beef cattle production and management course at Texas A&M University across five semesters (*N = *241; spring 2018 *= *61, summer 2018 *= *15, Fall 2018 *= *54, spring 2019 *= *55, and fall 2019 = 56). Male animal science students (*n *= 25; 32.9%) preferred assertive and direct communication (a driver communication style) and female students (*n *= 32; 19.4%) preferred collaborative and accommodating communication (an amiable communication style). Students were moderately experienced with beef cattle production (*M *= 3.09, *SD* = 1.07) before enrolling in the course; however, former beef cattle experiences did not influence their preferred communication style [*F*(10, 230) = 0.36, *P *= 0.96]. Researchers also observed students’ communication skills during an end-of-semester beef cattle production and management project presentation and identified strengths and weaknesses. Students demonstrated strong, in-depth animal industry knowledge, an ability to connect beef production techniques to management success, and critical thinking skills when answering questions. Oral communication skills warranting improvement included integrating visual aids and/or visual slides to support findings, using improved stage presence and confidence, and sharing responsibilities when presenting as a team. Finally, completion of a supplemental communication training module, intended to develop oral communication skills, significantly improved [*F*(1, 55) = 4.16, *P *= 0.046] students’ beef cattle production and management project presentation scores. As students become aware of their communication preferences and tendencies, they become equipped to adjust their communication practices and techniques when needed. Through this study, we gained insight into students’ communication tendencies and skills, which can be used to provide curricular recommendations and enhance students’ workforce readiness.

## Teaser

We identified the communication styles and communication effectiveness of upper-level, collegiate animal science students and, as a result, provided curricular and instructional recommendations that could improve students’ workforce development opportunities and communication preparation in the animal science industries.

## Introduction

Professionals who work in the animal-sourced foods industries are challenged with an increased need to communicate about the science of food production and processing (American Society of Animal Science, 2022b). A plethora of incomplete and inaccurate information is easily accessible to consumers, which leads to contradictory public views about animal science (Nicol, 2021), especially related to food animal welfare (Cornish et al., 2016; Alonso et al., 2020), environmental consequences (Rotz, 2020), food animal production practices (e.g., gene editing, growth regulators; Redding et al., 2021; Yunes et al., 2021), and healthfulness of food animal products (Drouillard, 2018). These contradictory public views pose challenges for enacting necessary improvements to food and agricultural systems and for food product developers and marketers (Macready et al., 2020). Therefore, there is a dire need for improved communication and education that builds trust between disconnected societal sectors and the public groups within them (Nicol, 2021).

Collectively, industry professionals are the drivers of information across multiple consumer groups (Parrish and Hamernik, 2015) who, as Burns et al. (2003) described, are “as multifaceted and unpredictable” as the individuals who identify as members of each respective group. Those similar, but unique, groups include mediators (e.g., communicators, educators, and opinion-makers), decision-makers, attentive public, interested public, and lay public (Burns et al., 2003). Though the groups are different, Rexroad et al. (2019) noted that the responsibility to communicate effectively about animal production across the public groups primarily falls to the next generation of animal scientists. However, a barrier to effective communication is that, on average, U.S. consumers are three generations removed from agriculture and have lesser knowledge and understanding of how food is produced compared to individuals who work in agriculture (Johnson and Hamernik, 2015). Similarly, many consumers rely on media reports and articles to make sense of animal agriculture information, causing further confusion and an increased risk of accessing misinformation (Verbeke, 2005; Sağlam and Gümüș, 2019). As a result, many next-generation animal scientists may not be adequately trained to communicate effectively, and many factors complicate the communication process.

Though communication is often convoluted, the sustainability of animal production partially depends on communication with public groups and social acceptability with public opinion (Capper and Yancey, 2015). Effective communication requires a commitment to building trust, shared values, strong ethics, and credible expertise (The Center of Food Integrity, 2015). This calls for animal scientists to be transparent in their dissemination of “truthful, objective, reliable, and complete” information (The Center of Food Integrity, 2015; Hake, 2020).

One way to create an environment for inclusive, transparent dialogue within the animal agriculture industry is to teach animal science students agricultural industry-specific communication skills and provide them opportunities to develop these context-specific skills that are foundational to their career success. Crawford et al. (2011) found that agricultural students at the undergraduate level, including those studying animal science, should develop communication skills related to listening effectively; communicating accurately, concisely, pleasantly, and professionally; communicating appropriately and professionally using social media; asking good questions; and communicating orally (CO) and in writing. Though these are key communication skills for career readiness, Harvey et al. (2020) later found that animal science students tasked with an assignment to create a brochure struggled to communicate concisely and tailor their message to a target audience.

Although many recognize more effective communication needs to occur, there is limited knowledge about students’ communication preferences and tendencies. This knowledge would better prepare educators to train students to communicate in the animal agriculture industry. Animal science students come from diverse backgrounds and enter college with a range of life experiences, all of which influence their success (Dunlap et al., 2019). One of these influential factors is agricultural involvement, like youth organization participation (e.g., 4-H and FFA). The historical foundation of 4-H and FFA provided youth with agricultural experiences to foster valuable life skills (e.g., communication, leadership, problem-solving, decision-making, teamwork, self-confidence, and interpersonal abilities; Holmgren and Reid, 2007; Kenderdine, 2019; Taylor, 2021). Similarly, youth with former beef cattle experiences often have unique responsibilities (e.g., tending to livestock daily, interacting with salespersons, and helping manage the business; Truax, 2020) that provide them valuable life skills critical to their career success.

Helping students identify and understand their preferred communication style has the potential to “help them become more effective communicators by deepening their understanding of the communication process” (Hartman and McCambridge, 2011). During the process of recognizing their preferred communication style, students recognized that others may have different communication styles and the interaction of these different styles was often the foundation of communication. This process of identifying personal and others’ communication styles is known as style-typing (Hartman and McCambridge, 2011), and it can help students strengthen the effectiveness of communication skills within their circle of influence.

### Purpose of study

To address the American Society of Animal Sciences’ (2022a) third core principle of communicating research and scientific information in an “open, transparent, and dynamic manner,” we investigated the communication styles and communication effectiveness of students enrolled in an upper-level, beef cattle production and management course at Texas A&M University. The following four research questions (RQ) and corresponding hypotheses (H) addressed our purpose.

RQ1: What is/are the preferred communication style(s) of animal science students?
H1.1: Animal science students exhibit a variety of communication styles.
RQ2: What effect does former beef cattle experience have on animal science students’ communication styles?
H2.1: Former beef cattle experience has a significant effect on animal science students’ communication styles.
RQ3: What communication skills are the most prominent in animal science students’ oral presentations?
H3.1: Animal science students display a variety of prominent communication skills in their oral presentations.
RQ4: Does supplemental training in communication skills influence animal science students’ beef cattle production and management presentation scores?
H4.1: Completing the communicating accurately and concisely (CAC) module has a significant effect on animal science students’ beef cattle production and management presentation scores.
H4.2: Completing the CO module has a significant effect on animal science students’ beef cattle production and management presentation scores.
RQ5: Does the time spent completing the supplemental training influence animal science students’ module scores?
H5.1: There is a significant relationship between time spent completing the modules and module scores.

## Materials and Methods

The study was a part of a federally funded project designed to develop and disseminate science communications curriculum for students studying animal, plant, and poultry sciences at American institutions. The current study did not require IACUC approval but did require IRB approval (IRB2017-0028M) before conducting human research. The results from the study described herein were included in a report submitted to the funding agency.

We used an explanatory sequential mixed methods design (Creswell and Creswell, 2018) that combined quantitative and qualitative data to gain a deeper comprehension of communication skill development in this beef cattle production and management course. We used a cross-sectional approach (Creswell and Creswell, 2018) for the descriptive study with four phases of data collection throughout five semesters. Cross-sectional studies allow researchers to investigate phenomena that influence a population at similar stages and specific points in time (Zheng, 2015). A cross-sectional design was most appropriate to achieve the study’s purpose because it provided us the opportunity to investigate variables influencing students’ communication skills in multiple semesters, which was necessary to gain a holistic perspective of communication strengths and needs in animal science students.

### Population

We selected students using a census approach (Creswell and Creswell, 2018). The study’s sample consisted of students enrolled in an elective, upper-level beef cattle production and management course at Texas A&M University throughout five semesters (*N = *241; spring 2018 *= *61, summer 2018 *= *15, fall 2018 *= *54, spring 2019 *= *55, and fall 2019 = 56). We omitted the summer 2019 semester because the primary researcher on the project had health complications and was unable to collect data. The study included 165 females (68%) and 76 males (32%). All (100%) students were senior (90 + completed credits) animal science majors.

The beef cattle production and management course is designed to address principles related to “profitable and sustainable, integrated beef cattle production” from the perspective of the “U.S. cow-calf sector, and, from an overall, systems-based approach” (Texas A&M University, 2018–2019). The course helps students:

Learn the fundamental concepts associated with cow-calf production, and how those concepts translate to other beef industry sectors and overall influences[;] gain knowledge related to coordinated management considerations for breeding, nutrition, and reproduction applications in beef cow herds[; and] understand the concepts affecting beef animal values and costs of production for improved marketing and profitability. (Texas A&M University, 2018–2019)

The course is listed as a communications-designated course, which is a classification of courses at Texas A&M University that warrant additional work and specific guidelines for writing assignments and oral presentations when compared to other 3- or 4-credit courses. Due to this communications designation, the course was ideal to investigate the communication styles and needs of upper-level animal science students.

We purposively selected the course to evaluate the capstone communication project where students organized, wrote, and presented a team-based beef cattle production and management project at the end of the semester. The final presentation, which included both oral and written components, was worth 15% of students’ course grades. Each group included four or five students who conducted an in-depth management plan for a Texas-based beef cattle production scenario using one of five scenarios per semester. The required sections for the management project are outlined in Table 1.

**Table 1.** Required sections and criteria for the beef production and management project

Required section^1^	Criteria to include
Introduction	Location details; beef cattle production and management plan scenario; owner profile; initial financial concerns/needs.
Topography	Types of grasses or forbs; soil type; average annual rainfall; water table and tributaries; predominant mineral deficiencies; concerns for the area.
Improvements	Fences needing repair; material needs and costs; pasture improvements; water improvements; facilities needed.
Production systems	Identified business system (e.g., purebred/seedstock or commercial production; selected breed or breed combinations; sire selection; calving and breeding considerations).
Animal health, nutrition	Mature cows; bulls; replacement heifers; weaning needs; and other cattle programs to include in the operation.
Marketing strategies	Target markets; target business goals.
Financial aspects	Annual operational budget; expenses and income projected on the total operation basis and individual sectors.
Overall recommendations	Air and GHG emissions; animal health and well-being; production efficiency and yield; employee safety; land resources; water resources; sustainability assessment report; summary.

^1^Beef cattle production and management course (ANSC 406) course syllabus, Texas A&M University, 2018–2019.

Instructors provided details about the management scenario during the third week of the semester. Subsequent lab activities and assignments supported the content of the project. Each group gave two, 5-min practice presentations during the semester to allow the instructors to provide formative feedback, and teams delivered the 15-min beef cattle production and management presentations via Microsoft PowerPoint during the second-to-last week of the semester. The instructor evaluated oral presentations based on the organization of content, thoroughness of information, presentation aspects (including communication delivery and media), and overall impression of the oral presentation. In addition, students completed individual written reports that described recommendations for the management plan 1 week before the oral presentations.

Interpersonal communication between team members also played a key role in the presentation scores as each student completed a confidential peer review of their team members and themselves, which could add or subtract up to 20 points (20%) of the final project grade. Students rated each member of their group, including themselves, on a scale from 0 to 10 (inferior to superior) based on four criteria: (1) overall level of participation; (2) contribution of ideas; (3) ability to draw useful information from sources, references, etc.; and (4) willingness to work. “Inferior evaluations” from team members could result in students receiving a grade of “F” for the project (Texas A&M University, 2018–2019). In summary, the integration of communication expectations in this course made the audience ideal for our study.

### Procedures

The study had four phases of data collection outlined by four RQs.

#### RQ1: preferred communication styles of animal science students.

We used Hartman and McCambridge’s (2011) communication style assessment to describe students’ communication styles as amiable, analytical, driver, and/or expressive using a survey design (Dillman et al., 2014). The communication style instrument (Hartman and McCambridge, 2011) consisted of 67 statements and required students to select if the statements described their communication style or not. Students wrote the number ‘1’ next to each statement that they believed described their communication style. Before completing the instrument, we asked students to consider their communication tendencies in team environments to gauge the style or styles they would most likely use to engage the audience during their beef cattle production and management presentations. After the assessment, students counted the number of ‘1s’ they placed next to the statements associated with each communication style, and the style with the largest number was the students’ dominant communication style in a team environment. Nineteen percent (*f *= 46) of students in the study had two or more dominant communication styles. We administered the instruments (Hartman and McCambridge, 2011) during the second week of the semester to capture students’ styles at the beginning of the course. We reported this ordinal data with means and standard deviations.

#### RQ2: effect of former beef cattle experience on students’ communication styles.

 We used survey methodology (Dillman et al., 2014) at the beginning of the semester to gauge students’ beef production experience levels before enrolling in the course (Appendix A). The course instructor administered these questionnaires during the first course meeting of the semester. The course instructor designed the beef cattle production and management experience questionnaire using a 5-point, Likert-type scale (1 = no beef cattle experience; 2 = limited experience (e.g., classes, field trips); 3 = moderate experience (e.g., 4-H or FFA projects for less than 5 years); 4 = experienced [e.g., worked cattle numerous times, many years of projects]; and 5 = very experienced [e.g., grew up and/or worked full time on cattle operation]). The instructor used these self-reported scores to separate students into teams for the management plan to balance those students with experience and those without. The instructor strived to balance experience as much as possible, but in some instances, teams had students with more former experience than others. We reported this ordinal data with means and standard deviations.

#### RQ3: communication skills prominent in beef cattle production and management oral presentations.

 We qualitatively assessed the strengths and weaknesses of students’ communication skills during the oral presentations. We observed the final team presentations and evaluated students’ communication skills using an oral presentation evaluation rubric that outlined the quality of students’ content (i.e., clarity and quality of content, originality and complexity of the project, the significance of the project, and support of main points); organization of content (i.e., appropriate use of media, smooth transitions between topics, logical flow of sections/ideas, clear thesis and supporting data, and informative and clear project summary); oral delivery (i.e., professionalism and confidence, engagement with an audience, clear voice, pace, command of language/avoidance of jargon, and response to questions); and overall impression and quality of oral presentation (Appendix B). At the end of the presentations, audience members (students also enrolled in the course) were expected to think critically about their peers’ presentations and ask meaningful questions. Teams answered five minutes of questions regarding their presentations.

Additionally, one of our team members evaluated students’ communication skills strengths and weaknesses during their final presentations. As a team, we sorted and analyzed the data by presentation teams and coded the data by hand using open and axial coding of the field notes to identify aggregate emergent themes, which is typical in qualitative research (Merriam and Tisdell, 2016). The oral presentation evaluation rubric provided a framework for key oral communication competencies displayed by the students. We assigned each team a number (i.e., T1 represented team 1), and we reported observations by teams (*N *= 61) rather than individual students across the five semesters. Our field notes were used only for the study described herein and were not used to evaluate students’ oral presentation scores or grades for the course. We analyzed all qualitative data by hand using open and axial coding of field notes to identify aggregate emergent themes (Merriam and Tisdell, 2016).

#### RQ4: supplemental communication training influence on beef cattle production and management presentation scores.

To meet the need for specialized communication skill training for agricultural science students, we developed supplemental communication modules as part of a USDA-funded project. These modules, outlined by Crawford et al.’s (2011) communication characteristics, included listening effectively, CAC, CO, communicating pleasantly and professionally, communicating in writing, asking effective questions, and communicating appropriately and professionally using social media.

To provide supplemental communication training for students in the course as they worked with their team to complete the comprehensive, summative beef cattle production and management project, we piloted two communication modules—CAC and CO—in the final semester of data collection for the current study, which was Fall 2019 (*N* = 56). The communication modules were hosted and accessed by students through Texas A&M University’s learning management system (Canvas; Instructure, Inc., Salt Lake City, UT), and students could complete these modules for extra credit in the course.

We investigated if completing the CAC and the CO modules influenced students’ beef cattle production and management presentation scores using a one-way, between-subjects ANOVA to compare the effect of completing the CAC and the CO modules on presentation scores between Spring 2018 and Fall 2019 at the *P *< 0.05 level. We met the assumption of homogeneity using Levene’s test (CAC, *P *= 0.51; CO, *P *= 0.51). We assessed the data using a boxplot and found no outliers in the data.

#### RQ5: time spent completing supplemental communication training influence on module scores.

 We used a simple linear regression to investigate the relationship between time spent completing the modules and the final presentation scores. We analyzed all quantitative data using IBM Statistical Package for the Social Sciences (SPSS) Statistics 28.0 for Macintosh.

## Results

The four RQs are provided below along with their results.

### RQ1: preferred communication styles of animal science students

We used Hartman and McCambridge’s (2011) communication style assessment to describe the communication styles of amiable, analytical, driver, and expressive for students enrolled across five semesters. Students preferred the amiable communication style (*n *= 80; 33.2%; Table 2). An additional 31 students (12.9%) identified as amiable in combination with a second communication style. Fifty-five students (22.8%) identified themselves as drivers alone, and 35 students (14.5%) identified as drivers in combination with another style. In the full study (*N *= 241) between spring 2018 and fall 2019, 165 students were female (68.5%). Females preferred the amiable (*n *= 58; 35.2%) communication style and males preferred driver (*n *= 25; 32.9%). Thirty-two females (19.4%) and 14 males (18.4%) had two or more dominant communication styles (Table 3).

**Table 2.** Students’ communication styles in a beef cattle production and management course at Texas A&M University across semesters

	Spring 2018		Summer 2018		Fall 2018		Spring 2019		Fall 2019		Overall	
Communication style^1^	*f* ^2^	%	*f* ^2^	%	*f* ^2^	%	*f* ^2^	%	*f* ^2^	%	*f* ^2^	%
Amiable	19	31.1	8	53.3	15	27.8	17	30.9	21	37.5	80	33.2
Driver	17	27.9	3	20	8	14.8	13	23.6	14	25	55	22.8
Analytical	9	14.8	—	0	14	25.9	6	10.9	12	21.4	41	17.0
Amiable/driver	9	14.8	—	0	5	9.3	4	7.3	2	3.6	20	8.3
Expressive	2	3.3	2	13.3	7	13	5	9.1	3	5.4	19	7.9
Analytical/driver	2	3.3	1	6.7	2	3.7	2	3.6	1	1.8	8	3.3
Driver/expressive	1	1.6	—	0	2	3.7	2	3.6	2	3.6	7	2.9
Amiable/analytical	2	3.3	1	6.7	1	1.9	2	3.6	—	0	6	2.5
Amiable/expressive	—	0	—	0	—	0	1	1.8	1	1.8	2	0.8
Amiable/analytical/Driver	—	0	—	0	—	0	2	3.6	—	0	2	0.8
Amiable/analytical/expressive	—	0	—	0	—	0	1	1.8	—	0	1	0.4

^1^The communication styles were based on Hartman and McCambridge’s (2011) communication style assessment that gauges four communication styles—driver communicators are typically more direct and assertive; amiable communicators are collaborative and accommodating; expressive communicators are relationship-driven and make quick decisions; and analytical communicators are task-driven and seek accuracy in messages, even if it takes longer to seek a solution. If students’ scores were equal for more than one communication style, we listed them as a double or triple communication style preference.

^2^ *f,* frequency.

**Table 3.** Students’ communication styles ranked by gender (*N = *241)

Communication style^1^	*f* ^2^	%
Female (*n *= 165)		
Amiable	58	35.2
Analytical	32	19.4
Driver	30	18.2
Amiable/driver	16	9.7
Expressive	13	7.9
Amiable/analytical	5	3.0
Driver/expressive	5	3.0
Analytical/driver	3	1.8
Amiable/expressive	2	1.2
Amiable/analytical/driver	1	0.6
Male (*n *= 76)		
Driver	25	32.9
Amiable	22	28.9
Analytical	9	11.8
Expressive	6	7.9
Analytical/driver	5	6.6
Amiable/driver	4	5.3
Driver/expressive	2	2.6
Amiable/analytical	1	1.3
Amiable/analytical/expressive	1	1.3
Amiable/analytical/driver	1	1.3

^1^Communication styles were based on Hartman and McCambridge’s (2011) communication style assessment that gauges four communication styles—driver communicators are typically more direct and assertive; amiable communicators are collaborative and accommodating; expressive communicators are relationship-driven and make quick decisions; and analytical communicators are task-driven and seek accuracy in messages, even if it takes longer to seek a solution. If students’ scores were equal for more than one communication style, we listed them as a double or triple communication style preference.

^2^ *f = *frequency.

### RQ2: effect of former beef cattle experience on students’ communication styles

Students across the five semesters were moderately experienced with beef cattle (*M *= 3.09, *SD* = 1.07 on a 1 to 5, lowest to highest scale; Table 4). These experience levels appeared consistent across study semesters. We found no relationship between former beef cattle experience and communication styles [*F*(10, 230) = 0.36, *P* = 0.96].

**Table 4.** Students’ former beef cattle production experience by semester (*N = *241)

Semester^1^	*Mean*	*SD*
Spring 2018 (*n *= 61)	3.02	1.01
Summer 2018 (*n *= 15)	3.33	1.23
Fall 2018 (*n *= 54)	3.06	1.11
Spring 2019 (*n *= 55)	3.15	1.10
Fall 2019 (*n *= 56)	3.09	1.07
Overall (*N *= 241)	3.09	1.07

^1^Five-point, Likert-type scale self-described as 1 = no beef cattle experience; 2 = limited experience (e.g., classes, field trips); 3 = moderate experience (e.g., 4-H or FFA projects for <5 yr); 4 = experienced (e.g., worked cattle numerous times, many years of projects); 5 = very experienced (e.g., grew up and/or worked full time on cattle operation).

### RQ3: communication skills prominent in beef cattle production and management oral presentations

We coded the qualitative findings by hand (Merriam and Tisdell, 2016) from the beef cattle production and management presentations by strengths and weaknesses of observed oral communication skills across the five semesters (Table 5).

**Table 5.** Summary and frequency of themes observed in the strengths and weaknesses of presentation teams’ communication skills (*N = *61)

Theme^1^	*Frequency*
Strengths	—
In-depth portrayal of animal industry knowledge	24
Ability to connect beef production techniques to management success	5
Critical thinking skills when answering questions	8
Weaknesses	—
Incorporate visual aids and/or visual slides to support findings	18
Improve stage presence and confidence	22
Share responsibilities when presenting as a team	14

^1^Frequencies include the number of occurrences of each theme. We hand-coded the data using open and axial coding of the field notes to identify aggregate emergent themes (Merriam and Tisdell, 2016). Some teams expressed exemplary statements comprised of more than one theme.

#### Oral communication strengths.

Student’s strengths in oral communication included *an in-depth portrayal of animal industry knowledge, an ability to connect production considerations to management success*, and *the use of critical thinking skills when answering questions*.

First, teams holistically demonstrated an in-depth portrayal of animal industry knowledge in beef management plans. Throughout the course, students learned strategies to establish practical and efficient production systems based on the needs of the assigned management scenario. Teams demonstrated mastery of terminology and reasoning to support their decisions for when to use different production systems, animal health plans, and nutritional goals. For example, to diversify their portfolio, one team incorporated cow/calf and stocker operations. In their presentation, they discussed confidently and accurately their plan for distributing salt and commercial minerals, managing the existing forage, and introducing new pasture management techniques suitable for their area. Furthermore, many teams explained the improvement of genetics in operations by introducing bulls with lower birth weights in locations with more arid climates and maximizing heterosis through implementing crossbred programs.

Teams also demonstrated a strong ability to connect production considerations to management success. Instructors required students to outline the plan’s strengths and opportunities (e.g., topography, climate, stocking rate, existing production assets and structures, and established genetics) at the beginning of the presentation, which guided several teams to describe clearly why they chose their production systems, breeding programs, nutritional needs, etc. Although each management scenario was unique, several teams provided specific recommendations to improve the bottom line of their respective operations through more efficient breeding programs, adjusting herd size to meet the grazing requirements of different pastures, and implementing improved grazing practices.

One team used a creative approach to increase the financials of the hypothetical operation. In addition to establishing a Charolais seedstock scenario, the team incorporated a quail hunting enterprise and conservation plan to improve wildlife habitat and native grasses to diversify their business ventures and addressed the impact of each diversified business venture through the summary of their presentation on the final slide. Another team also suggested a diversified approach to its production by adding entertainment features to difficult-to-graze pastures. Supported by being located near an urban setting, the team designed a paintball course in a thick, wooded pine area near the back of their scenario’s property and designed a marketing strategy to lure people from the urban location for a paintball adventure.

Finally, teams showcased a strong aptitude for using critical thinking skills when answering questions from instructors and peers. At the end of the presentations, teams answered 5 min of questions related to their presentations. Several teams provided thoughtful answers that demonstrated comprehensive critical thinking skills. Many audience members asked thoughtful questions about herd health and vaccination plans. One team eloquently explained their reasoning for implementing a 7-way, clostridial vaccine with a modified-live virus to protect against a bovine respiratory disease for their calves at 2.5 mo followed by a booster vaccination at weaning. Several teams answered questions regarding marketing strategies, and many teams shined when they highlighted their plans for social media outreach, website design, and the creation of branded materials for their beef cattle operation. When asked about other unique ways to connect to their audience and promote the brand, one team suggested creating promotional materials to promote genetics to livestock youth at county, state, and national fairs.

#### Oral communication weaknesses.

Based on the beef cattle production and management presentations, the oral communication skills that evaluators determined needed improvement included *incorporating visual aids and/or visual slides to support findings*, *improving stage presence and confidence*, and *sharing responsibilities when presenting as a team*.

First, the oral communication skill that needed the most improvement across the five semesters was the need to *incorporate visual aids and/or visual slides to support findings*. The instructor required all teams to present their management plans to their peers using a PowerPoint presentation. Several teams placed multiple bullets in small fonts with full lines of text on each slide that were often difficult for the audience to read. Some teams also used the slides as content and read the text word-for-word rather than as a supplement to the presentation. When reading the text from the slides, several students also used this as a crutch for not maintaining appropriate eye contact with the audience. In some situations, the slides only contained paragraphs of text with no visual element other than text. Occasionally, the teams would include stock photo visual aids pulled from the internet but would not select stock photos that directly matched their production facility. For example, one team used several pictures of Hereford cows grazing when the management plan was for an Angus cattle operation.

Second, across the five semesters, students needed to *improve stage presence and confidence*. Confidence levels varied across teams where some students were clearly more comfortable speaking in front of an audience than other students as indicated by their voice, volume, eye contact, and mannerisms. In many situations, students who appeared more confident in their presentations likely had former beef cattle experience before taking the course as they would include their former experience in their presentations and questions. Some mannerisms displayed by students that reflected nervousness or a lack of confidence included rocking back and forth; fidgeting with hands, pens, or the slide advancer; lacking eye contact when reading directly from the slides or staring at the ground; or using crutch words, such as “umm,” “uh,” “like,” or “so.”

Finally, students could improve their ability to *share responsibilities when presenting as a team.* Team projects often create a unique dynamic when students with differing communication styles, goals, and time commitments work together. However, several teams included students who clearly presented more details and understood the scope of the project more than other students. This characteristic was especially true while answering questions from the audience. Certainly, students faced a time restraint when answering questions; even still, in many cases, one or two students from each team were the only ones who answered questions even though, in some cases, they were not the ones who presented the content related to the question.

### RQ4: supplemental communication training influence on beef cattle production and management scores

There was no difference between students who completed the CAC module and those who did not [*F*(1, 55) = 2.55, *P *= 0.116]. However, we did find a difference between students who completed the CO module and those who did not [*F*(1, 55) = 4.16, *P *= 0.046]. Students who completed the CO module received a higher grade (*P *= 0.046) on the management presentations.

### RQ5: time spent completing supplemental communication training influence on module scores

Twenty-three students (41%) completed at least one optional module, and students averaged 32 min and 52 s (*SD = *21 min and 58 s) to complete each module. Of these voluntary participants, 75% were female (*n *= 17) and 25% were male (*n *= 6). Results of a simple linear regression indicate that time spent completing the modules was not related to module scores (*β* = −0.01, *P* < 0.841).

## Discussion

Just as Hartman and McCambridge (2011) suggested, communication competence can have a direct link to employment success and satisfaction. By understanding their communication preferences and tendencies, students can learn to accommodate others during the communication process. With practice and style-flexing, students can learn to recognize and predict the preferred communication styles of others and modify their personal preferences and tendencies to improve their communication effectiveness (e.g., consider the wording of questions they ask, adjust their pace to match that of their counterpart; Hartman and McCambridge, 2011). Therefore, awareness of communication styles does not only support communication preparation but can also reinforce workforce development as students seek to communicate complicated scientific topics (e.g., ruminant nutrition and management) to lay audiences.

Investigating animal science students’ primary communication styles can help faculty and staff tailor communication training (e.g., curriculum and workshops) and communication opportunities (e.g., activities and assignments). For example, animal science students in the study tended to prefer amiable and driver communication styles. Because amiable communicators are generally accommodating and less assertive, we recommend implementing decision-making exercises to help these students become more decisive communicators. Driver communicators, however, tend to be assertive and unresponsive to differing viewpoints. These communication tendencies can be especially problematic for science communicators who need to engage with lay audiences about complex and polarized agricultural topics, like livestock production. Therefore, we recommend to (1) identify students’ communication styles at the beginning of each course and, (2) implement empathy-building exercises (e.g., role-play) to help students develop their ability to acknowledge, respect, and respond appropriately to counterparts who think differently than themselves.

The animal science students in the study’s sample generally had moderate beef cattle experience, which was less than some animal science programs at land grant institutions (e.g., the University of Nebraska-Lincoln where the majority of whom were 4-H and FFA members and grew up on a farm or ranch; Winkel et al., 2019). Goodwin et al. (2005), Rose et al. (2016), Freeman (2017), and Truax (2020) all found that former beef cattle experience significantly affected agriculture students’ development of life skills (e.g., self-esteem, time management, and leadership). However, we found that animal science students’ beef cattle experience had no effect on their preferred communication styles. We recommend future research to investigate if relationships between previous beef management experience influence animal science students’ communication skill development—an outcome we did not consider. Future research should explore other factors unique to animal science students that may influence their preferred communication styles, such as amount of animal-related internship or work experiences, participation on a collegiate judging team, and extent of science communication involvement (Winkel et al., 2019).

Furthermore, we directly observed three communication strengths—in-depth portrayal of animal industry knowledge; ability to connect beef production techniques to management success; critical thinking skills when answering questions—of Texas A&M University animal science students during their beef cattle production and management presentations. Students’ in-depth industry knowledge will help them build trust, demonstrate credible expertise, and share complete and accurate information with consumers (The Center of Food Integrity, 2015). Crawford et al. (2011) emphasized the need for students to ask meaningful questions, and we believe student’s ability to apply critical thinking skills when answering questions—a strength we observed—is equally as important.

We also observed three communication weaknesses of these animal science students—need to incorporate visual aids and/or visual slides to support findings; improve stage presence and confidence; and share responsibilities when presenting as a team. Proper visual aid usage can help animal scientists demonstrate stronger oral communication skills by visually tailoring messages to a target audience (Barry and Orth, 2013; Robinson and Mulvaney, 2018), which is a strategy that animal science students have struggled to achieve (Harvey et al., 2020). Visual aids can also enable animal scientists to communicate shared values and transparency (The Center of Food Integrity, 2015; Hake, 2020). Students in this study would have benefited from additional training related to designing effective visual media in their presentations.

Students in our sample needed more opportunities to improve their stage presence, which could directly improve their ability to communicate (Crawford et al., 2011). Public speaking confidence can also help animal scientists demonstrate credible expertise and improve their oral communication skills, especially during delivery (Crawford et al., 2011; Barry and Orth, 2013; The Center of Food Integrity, 2015; Robinson and Mulvaney, 2018). Furthermore, students must learn to share responsibilities when presenting as a team, which often comes with practice and a desire to contribute. As such, animal science students benefit from guidance and opportunities to develop their team presentation skills, which can improve their ability to display professionalism (Crawford et al., 2011).

Moreover, supplemental communication skill training, specifically completion of the CO module, significantly improved animal science students’ management presentation scores. This finding warrants additional longitudinal research investigating if exposure to supplemental communication training has a long-term impact on students’ ability to connect with lay audiences and consumers. Additionally, pilot studies indicated that each module should last 1 to 2 h. Because most students completed these modules using nearly half of the expected time, they might not have retained content. This could explain why the CAC module did not affect students’ scores. Therefore, we recommend instructors incorporate supplemental communication training into their courses and consider assigning a grade for completion rather than considering it as optional. Assigning a grade would likely increase the number of students who complete the training and increase their attention to the material included in the training. It may also be interesting to gauge if participants who completed the modules retained information, and to seek post-reflection feedback from those students to gauge how they rate the impact of the modules.

This study is the first to investigate the communication styles and communication effectiveness of upper-level animal science students. Although our results are only generalizable to students who took the beef cattle production and management course at Texas A&M University, our recommendations might benefit similar students elsewhere. Importantly, future research should examine the same characteristics of animal science students at other universities, and results should be compared across programs. Together, these assessments could inform the development of an evidence-based interdisciplinary curriculum designed to improve animal science students’ communication skills and be implemented on a large scale. To access the communication skills modules (Leggette and Murphrey, 2023) designed for students studying animal science at the college level, visit https://scicomm.tamu.edu/home/science-communications-curriculum/.

## Supplementary Material

txae007_suppl_Supplementary_Appendix_A

txae007_suppl_Supplementary_Appendix_B

## Abbreviations

4-H: head, heart, hands, and health
ASAS: American Society of Animal Science
CAC: communicating accurately and concisely
CO: communicating orally
FFA: National Future Farmers of America Organization
IACUC: Institutional Animal Care and Use Committee
IRB: Institutional Review Board
NACTA: North American Colleges and Teachers of Agriculture
USDA: United States Department of Agriculture
