# Supplementary material for: Beefing up communication skills of upper-level animal science students
Source: Transl Anim Sci. 2024 Jan 12;8:txae007. doi: 10.1093/tas/txae007 (PMC10836500; doi:10.1093/tas/txae007)
Supplement: txae007_suppl_Supplementary_Appendix_A [file txae007_suppl_supplementary_appendix_a.pdf]

**ANSC 406**
**Texas A&M University**
**Beef Cattle Production & Management**
**Background Information**

**Name (preferred name):**

**Major:**

**Hometown:**

**Experience with farm animals (please be specific):**

Circle one (only one) of the choices below that best describes your experiences with beef cattle:

| 1 | 2 | 3 | 4 | 5 |
|---|---|---|---|---|
| **None** | **Limited** (classes, field trips, etc. only) | **Moderate** (4-H, FFA projects, etc. only for less than 5 years) | **Experienced** (Worked cattle numerous times, many years of projects, etc.) | **Very experienced** (grew up and/or worked full time on cattle operation) |

What kind of knowledge do you wish to gain from this class (i.e. what areas about beef cattle production do you want to know more about)?

Some <u>possible</u> career paths for you (i.e. what would you like to get paid for) might include:

Which of these (or similar) courses have you completed, or are currently enrolled?

ANSC 303 (Nutrition) or ANSC 318 (Feeds and Feeding) ______________
ANSC 333 (Reproduction in Farm Animals) ______________
ANSC 305 (Animal Breeding) ______________
