# Supplementary material for: Beefing up communication skills of upper-level animal science students
Source: Transl Anim Sci. 2024 Jan 12;8:txae007. doi: 10.1093/tas/txae007 (PMC10836500; doi:10.1093/tas/txae007)
Supplement: txae007_suppl_Supplementary_Appendix_B [file txae007_suppl_supplementary_appendix_b.pdf]

ORAL PRESENTATION EVALUATION FORM

PLEASE NOTE: THIS FORM WILL BE GIVEN TO THE PRESENTER AFTER THE EVENT TO PROVIDE FEEDBACK

Name of Presenter: ______________________ Department / School: ______________________

Platform Session: ______________________________________

Please mark the score for each evaluation criterion below. When you are finished, combine the total points at the bottom for the overall score.

	Poor	Fair	Average	Good	Excellent
**Content**					
Clarity of content	1	2	3	4	5
Quality of content (background, methodology, findings, etc.)	1	2	3	4	5
Originality and complexity of project	1	2	3	4	5
Significance of project (to field of study, community, etc.)	1	2	3	4	5
Support main points	1	2	3	4	5

Comments on Content:

Content Points = _____ / 25

	Poor	Fair	Average	Good	Excellent
**Organization**					
Appropriate use of media	1	2	3	4	5
Smooth transitions between topics	1	2	3	4	5
Logical flow of sections/ideas	1	2	3	4	5
Clear thesis and supporting data	1	2	3	4	5
Informative and clear project summary	1	2	3	4	5

Comments on Organization:

Organization Points = _____ / 25

	Poor	Fair	Average	Good	Excellent
**Delivery**					
Professional and confident	1	2	3	4	5
Engaged with audience	1	2	3	4	5
Clear voice with good pace	1	2	3	4	5
Command of language/avoiding jargon	1	2	3	4	5
Response to questions	1	2	3	4	5

Comments on Delivery:

Delivery Points = _____ / 25

	Poor	Fair	Average	Good	Excellent
**Overall Impression/Quality**	1	2	3	4	5

Overall Impression Points = _____ / 5

Comments *(may use back of paper as well)*

TOTAL SCORE = ______ / 80

What were the strengths of this presentation?

Do you have any suggestions for improvement?
